# Ferulic Acid Alleviates Changes in a Rat Model of Metabolic Syndrome Induced by High-Carbohydrate, High-Fat Diet

**DOI:** 10.3390/nu7085283

**Published:** 2015-08-04

**Authors:** Ketmanee Senaphan, Upa Kukongviriyapan, Weerapon Sangartit, Poungrat Pakdeechote, Patchareewan Pannangpetch, Parichat Prachaney, Stephen E. Greenwald, Veerapol Kukongviriyapan

**Affiliations:** 1 Department of Physiology, Faculty of Medicine, Khon Kaen University, Khon Kaen 40002, Thailand; E-Mails: ketmanee.879@gmail.com (K.S.); weerapons@kkumail.com (W.S.); ppoung@kku.ac.th (P.P.); 2 Department of Pharmacology, Faculty of Medicine, Khon Kaen University, Khon Kaen 40002, Thailand; E-Mails: patc_pan@kku.ac.th (P.P.); veerapol@kku.ac.th (V.K.); 3 Department of Anatomy, Faculty of Medicine, Khon Kaen University, Khon Kaen 40002, Thailand; E-Mail: parpra@kku.ac.th; 4 Blizard Institute, Barts and The London School of Medicine and Dentistry, Queen Mary University of London, London E1 2ES, UK; E-Mail: s.e.greenwald@qmul.ac.uk

**Keywords:** endothelial dysfunction, ferulic acid, high-carbohydrate-high-fat diet, inflammation, metabolic syndrome, oxidative stress, vascular remodeling

## Abstract

Metabolic syndrome is a cluster of metabolic abnormalities characterized by obesity, insulin resistance, hypertension and dyslipidemia. Ferulic acid (FA) is the major phenolic compound found in rice oil and various fruits and vegetables. In this study, we examined the beneficial effects of FA in minimizing insulin resistance, vascular dysfunction and remodeling in a rat model of high-carbohydrate, high-fat diet-induced metabolic changes, which is regarded as an analogue of metabolic syndrome (MS) in man. Male Sprague-Dawley rats were fed a high carbohydrate, high fat (HCHF) diet and 15% fructose in drinking water for 16 weeks, where control rats were fed with standard chow diet and tap water. FA (30 or 60 mg/kg) was orally administered to the HCHF and control rats during the last six weeks of the study. We observed that FA significantly improved insulin sensitivity and lipid profiles, and reduced elevated blood pressure, compared to untreated controls (*p* < 0.05). Moreover, FA also improved vascular function and prevented vascular remodeling of mesenteric arteries. The effects of FA in HCHF-induced MS may be realized through suppression of oxidative stress by down-regulation of p47phox, increased nitric oxide (NO) bioavailability with up-regulation of endothelial nitric oxide synthase (eNOS) and suppression of tumor necrosis factor-α (TNF-α). Our results suggest that supplementation of FA may have health benefits by minimizing the cardiovascular complications of MS and alleviating its symptoms.

## 1. Introduction

Plant polyphenols are phytochemical compounds found in various plants and fruits. This group of compounds has been intensively investigated as a potential source for treatment of various diseases including metabolic syndrome, diabetes and cancer [1]. Polyphenolic compounds are classified into simple phenols, flavonoids, hydroxycinnamic acids, coumarins, xanthones, acetophenones, phenylacetic acids and the less common stibenes and lignans [2,3]. These natural polyphenols have been shown to have varying bioavailability and marked health benefits in various diseases [1]. Ferulic acid (FA; 4-hydroxy-3-methoxycinnamic acid), a hydroxycinnamic acid derivative, is abundant in fruits and vegetables, such as tomato, orange, other citrus fruits, carrot, sweet corn, cabbage, broccoli, banana and rice bran [4,5]. FA is esterified in various forms in these sources. It is relatively well absorbed when compared with flavonoid compounds [6]. Numerous studies have shown that FA possesses potent antioxidant activity by scavenging free radicals and enhancing the cell stress response through the up-regulation of the cytoprotective system [7]. Moreover, FA has been shown to reduce systolic blood pressure in spontaneously hypertensive rats (SHR) [8], and to elicit improved endothelial function in 2 kidney-1 clip (2K-1C) hypertensive rats and high fat diet rabbits [9,10]. Treatment with FA decreased blood glucose in mice [11], reduced plasma triglyceride, free fatty acid and total cholesterol in diabetic rats and mice [12,13]. FA also decreases some inflammatory mediators such as prostaglandin E2 and tumor necrosis factor-alpha (TNF-α) [14], improves nitric oxide (NO) bioavailability and increases NO synthesis [9]. Based on this evidence, FA may offer beneficial effects against many disorders associated with oxidative stress and inflammation including metabolic syndrome, diabetes, cardiovascular disease, Alzheimer’s disease and cancer [5,7].

Metabolic syndrome is a major health problem which predisposes those affected to the development of type 2 diabetes, cardiovascular and kidney diseases [15]. It is characterized by the presence of three or more of the following risk factors: hypertension, hyperglycemia, dyslipidemia, obesity and insulin resistance [15]. The prevalence of metabolic syndrome is rapidly increasing worldwide including the developing countries. This is due primarily to prevailing sedentary lifestyles and unhealthy dietary habits [16], specifically a diet rich in saturated fat and carbohydrates such as fructose and sucrose. Intake of this diet is associated with many complications including cardiovascular disease, nonalcoholic fatty liver disease (NAFLD) and metabolic syndrome [17].

Although the pathogenesis of metabolic syndrome is complex and the underlying mechanisms are not clearly understood, many experimental animal models for metabolic syndrome have enriched our understanding of the etiology, pathophysiological basis and the development of therapies as described in the literature [18,19,20]. Data obtained from various studies has shown that the animal models of metabolic syndrome mimic the major signs of metabolic syndrome in humans, especially hypertension, dyslipidemia, diabetes, impaired glucose tolerance, obesity and insulin resistance. Among all these animal models, the evidence suggests that chronic consumption of a high-carbohydrate (in the form of fructose) and high-fat diet by normal rodents comes the closest to fulfilling the criteria by which metabolic syndrome in man are defined [20]. Accordingly, in what follows, the abbreviation (metabolic syndrome (MS)) should be taken to denote the high carbohydrate, high fat (HCHF) rat model of MS. Previous studies have demonstrated that rats fed with high carbohydrate and high fat diet for few months develop as revealed by insulin resistance, dyslipidemia, vascular dysfunction, inflammation, fibrosis and enlargement of the heart with structural remodeling [17,21]. Compounds that could prevent MS and long-term vascular complications, such as vascular remodeling, could be very beneficial for health promotion.

Impaired vascular function is probably associated witha diminution of the vasoprotective effect of endothelial NO and increased oxidant stress by enhanced formation of reactive oxygen species (ROS) and release of pro-inflammatory mediators (e.g., TNF-α) [14,22]. The aim of this study was to determine whether FA could prevent metabolic syndrome and vascular remodeling in rats in which MS was induced by an HCHF diet, and to elucidate the mechanism underlying the alleviation of oxidative stress, inflammation and vascular dysfunction.

## 2. Experimental Section

### 2.1. Animals and Diets

Male Sprague-Dawley rats weighing 220–250 g were supplied by the National Laboratory weighing Center, Mahidol University, Salaya (Nakornpathom, Thailand). After 7 days of acclimatization, the rats were randomly assigned to 2 groups: a control group (C, *n* = 32) received standard rat chow diet (Chareon Pokapan Co. Ltd., Bangkok, Thailand) with tap water; and a high-carbohydrate, high-fat diet group (HCHF; *n* = 48), which were fed with HCHF diet together with 15% fructose in drinking water for 16 weeks. At week 10, the metabolic syndrome state was confirmed by measurement of fasting blood glucose (FBG) (≥100 mg/dL), systolic blood pressure (SBP) (≥140 mmHg) and lipid profiles (hypertriglyceridemia or low high density lipoprotein-cholesterol (HDL-C) level). All HCHF rats that satisfied the presumptive MS criteria were randomly divided into 3 groups (*n* = 16/group) with matched body weight, SBP, FBG and lipid profiles. The studied groups were treated as follows for the last 6 weeks of the experimental period: (1) Normal control rats were divided into 2 groups of 16. The first (group 1) was treated orally with vehicle alone, propylene glycol (PG), at 1.5 mL/kg/day: (C + PG), the second control group (2) was treated orally with ferulic acid (FA) 60 mg/kg/day: (C + FA60). The first of the three groups of the HCHF rat model of MS (3) was treated orally with PG, vehicle at 1.5 mL/kg/day: (MS + PG), the second MS group (4) was treated orally with a FA (30 mg/kg/day): (MS + FA30); and the third MS group, (5) was treated orally with a high dose of FA (60 mg/kg/day): (MS + FA60).

**Figure 1:**
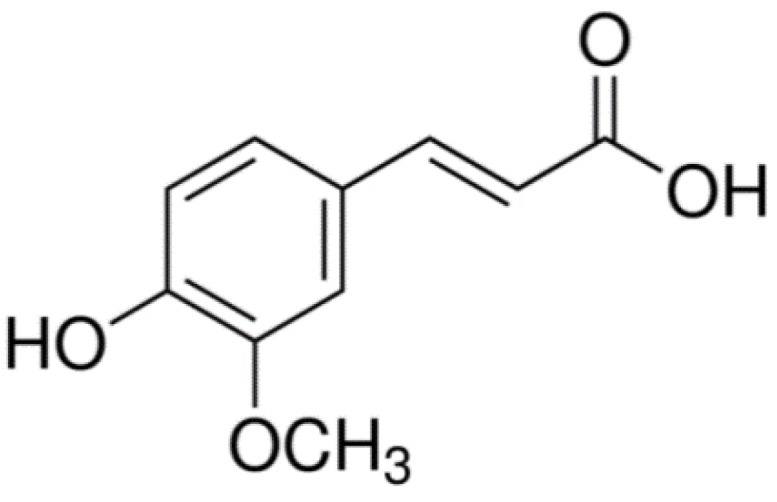
Ferulic acid.

Ferulic acid (FA; *trans*-Ferulic acid 99%) (Figure 1) was obtained from Sigma-Aldrich (St. Louis, MO, USA) (Figure 1). All the rats were housed at the Northeast Laboratory Animal Center (Khon Kaen University, Khon Kaen, Thailand) in a temperature-controlled (25 ± 2 °C) room, on a 12h light/dark cycle with free access to the group-specific diets and water. All experimental protocols were approved by the Animal Ethics Committee of Khon Kaen University.

The composition and preparation of the HCHF diet has been described in a previous study [17] with some modifications. It consisted of 175 g fructose, 350 g condensed milk, 200 g pork tallow, 200 g powdered rat chow, 25 g of Hubble, Mendel and Wakeman salt mixture, and 50 g water per kilogram of diet. The energy densities in the food pellets of control and HCHF feeding diets are shown in Table 1. As the key carbohydrate for the HCHF group is fructose, the drinking water for this group was supplemented with 15% fructose. Meanwhile, the standard chow-fed rats received tap water. Therefore, animals in the HCHF group received more carbohydrate than those in the control group. Rats in all groups were given free access to food and water.

**Table 1:** Energy densities in the food pellets of control and MS groups.

Macronutrient Composition	Standard Chow Diet	HCHF Diet
**Total carbohydrate, g/100g**	56.24	55.06
**Total fat, g/100g**	5.78	18.85
**protein, g/100g**	24.76	8.83
**Crude fiber, g/100g**	1.91	0.75
**Ash, g/100g**	6.12	3.94
**Moisture, g/100g**	4.40	13.82
**Energy, Kcal/100g**	386.82	423.21

MS, a rat model of metabolic syndrome; HCHF, high carbohyrate and high fat.

### 2.2. Physiological and Metabolic Variables

All rats were monitored for diet consumption and water intake. The weight gain of each rat was measured weekly. Plasma concentrations of total cholesterol, triglyceride (TG) and HDL-cholesterol were monitored before the feeding period (*i.e*., at the end of the 7 day acclimatization period), at week 10 and at the end of experimental period by using a timed-endpoint methods [23].

### 2.3. Indirect Measurement of Blood Pressure in Conscious Rats

The SBP was monitored every 4 weeks in conscious rats, pre-warmed (32 °C) for 10 min by non-invasive tail-cuff plethysmography (IITC/Life Science, Woodland Hills, CA, USA). Five repeated measurements were taken for each rat. The individual SBPs were obtained from an average of 3 consistent readings of SBP. After 8 weeks, SBP was measured every 2 weeks until the end of experimental period.

### 2.4. Fasting Blood Glucose, Oral Glucose Tolerance Test

An oral glucose tolerance test (OGTT) was performed every 4 weeks and FBG was measured every 2 weeks starting before the feeding period. Blood samples were taken from a lateral tail vein to measure FBG using a glucometer (Roche Diagnostics, Sydney, Australia). After 8 weeks, the OGTT was measured every 2 weeks throughout the last 8 weeks of experimental period. Before the OGGT, rats were deprived of diet for 8–12 h. And the 15% fructose-supplement drinking water in the HCHF group was replaced by normal drinking water during this period. The rats were subjected to a glucose load of 2 g/kg body weight (orally administered) and blood glucose concentrations were measured before glucose loading and at 30, 60, and 120 min after administration. Blood glucose concentrations over the period of 120 min were used to calculate the area under the curve (AUC) of the concentration time curve.

### 2.5. Fasting Serum Insulin Assessments and Homeostasis Model Assessment-Estimated Insulin Resistance (HOMA-IR) Calculation

Fasting serum insulin concentrations was measured at the end of experimental period using a Rat Insulin enzyme-linked immunosorbent assay (ELISA) Kit (Millipore, Billerica, MA, USA). HOMA-IR score, an index of insulin resistance [24] was calculated using Expression (1):

HOMA = (Fasting glucose (mmol/L) × Fasting insulin (µlU/mL))/22.5
(1)

### 2.6. Hemodynamic Measurements

At the end of the 16 week experimental period, all rats were anesthetized with an intraperitoneal injection of pentobarbital sodium (60 mg/kg). The femoral artery was cannulated with a polyethylene tube and connected to a pressure transducer for monitoring blood pressure (BP) and heart rate (HR). Hindlimb blood flow (HBF) and hindlimb vascular resistance (HVR) were also measured, as previously described [25]. After blood flow measurements, vascular reactivity was evaluated by infusing vasoactive agents at various doses through an additional catheter in the femoral vein in a stepwise fashion at 5-min intervals. The vasoactive agents tested were an endothelial-dependent vasodilator, acetylcholine, (ACh; 3, 10, 30 nmol/kg) [26], an endothelial-independent vasodilator, sodium nitroprusside (SNP; 1, 3, 10 nmol/kg) [26], and an alpha sympathomimetic agent, phenylephrine (Phe; 0.01, 0.03, 0.1 µmol/kg) [27]. Changes in blood pressure were expressed as percentage of control values obtained immediately before the administration of the test substance. After hemodynamic measurements, rats were sacrificed with an overdose of the anesthetic drug. Blood samples were collected from the abdominal aorta and centrifuged at 3500× *g* for 15 min at 4 °C to obtain the plasma for assaying plasma TNF-α and oxidative stress markers. Heart, left ventricle (LV) and liver were separated and weighted. Organ weights were normalized with respect to body weight (mg/g body weight (BW)). The aorta and carotid arteries were rapidly isolated from the rats and used for Western blot analysis of endothelial nitric oxide synthase (eNOS) and p47^phox^ nicotinamide adenine dinucleotide phosphate (NADPH) oxidase expression, and superoxide production (O_2_^•−^).

### 2.7. Oxidative Stress and Inflammation

#### 2.7.1. Superoxide Production and Plasma MDA

Assessment of vascular superoxide (O_2_^•−^) production was performed in isolated carotid arteries using a lucigenin-enhanced chemiluminescence technique as previously reported [25,26]. Plasma malondialdehyde concentrations (MDA) were determined by measuring thiobarbituric acid reactive substances following a previously described method [28].

#### 2.7.2. Assay of Nitric Oxide Metabolites

The levels of nitrate/nitrite in the plasma, the end products of NO metabolism, were quantified by an enzymatic conversion method with the Griess reaction as previously described [26].

#### 2.7.3. Plasma TNF-α

The plasma concentration of TNF-α was determined by an ELISA kit (eBioscience, San Diego, CA, USA).

### 2.8. Histology

Arteries from six rats of each group were used for histology. At the end of the experiment, rats were sacrificed with an overdose of pentobarbital sodium, mesenteric resistance arteries were collected and fixed with 4% phosphate-buffered formaldehyde. To determine medial cross-sectional area (CSA), arterial wall thickness and media to lumen ratio (M/L), the mesenteric arteries were embedded in paraffin blocks and 5 µm thick sections were cut and stained with hematoxylin and eosin (H&E). The stained sections were examined with light microscopy (Nikon ECLIPSE Ni-u, Nikon Instruments Inc., Melville, NY, USA) and the images were captured with a digital microscope camera (Nikon DS-Ri1 Camera). CSA, measured in tissue sections under a ×40 objective was calculated by subtractingthe lumen area (A_i_) from the total vessel area including the lumen (A_e_). The external radius (R_e_) and the internal radius (R_i_) were calculated as the square root of A_e_/π and A_i_/π, respectively. Arterial wall thickness was calculated as R_e_ minus R_i_. Finally, M/L ratio was calculated as the wall thickness divided by radius of the lumen [25].

### 2.9. Western Blot Analysis

Western blotting was performed in aortas from each experimental group to detect eNOS and p47^phox^ as previously described [25,28]. Briefly, proteins of aortic homogenates were separated by electrophoresis on 10% sodium dodecyl sulfate polyacrylamide gel. The proteins were electrophoretically transferred to a polyvinylidene difluoride membrane, blocked with 5% skimmed milk in Tris buffered saline containing 0.1% Tween-20 and then incubated with primary antibody of mouse monoclonal anti-eNOS (BD Bioscience, San Jose, CA, USA) and mouse monoclonal anti-p47^phox^ (Santa Cruz Biotechnology, Indian Gulch, CA, USA) overnight. Then membranes were repeatedly washed and incubated with the secondary antibody horseradish peroxidase goat anti-mouse immunoglobulin G (IgG) (Santa Cruz Biotechnology) for 2 h at room temperature. The blots were incubated in the enhanced chemiluminescent (ECL) substrate solution (Thermo Fisher Scientific, Rockford, IL, USA). The intensity of specific eNOS, p47^phox^ and β-actin bands was visualized and captured by ImageQuant™ 400 (GE Healthcare Life Science, Pittsburgh, PA, USA). The expression of eNOS, p47^phox^ protein wasnormalized to β-actin expression from the same sample and values are presented as percentages of those from the aorta of normal controls.

### 2.10. Statistical Analysis

All data are presented as mean ± standard error of the mean (SEM). Statistically significant differences among groups were calculated using one way analysis of variance (ANOVA) followed by the Student-Newman-Keuls *post hoc* test. Statistical significance was defined as *p* < 0.05.

## 3. Results

### 3.1. Effect of FA on Body Weight and Organ Weight of MS Rats

Rats fed with HCHF gained weight at a similar rate to control animals on the normal diet and supplementation with FA in the normal diet or HCHF diet groups did not affect body weight (Table 2). However, MS rats showed a marked increase in liver weight and a small increase in heart and left ventricular weight when compared with control rats. Treatment with FA apparently normalized the increased liver weight, while FA did not alter organ weight in normal rats (Table 2).

**Table 2:** Effect of FA on body weight and organ weight in all experimental groups.

Variables	C + PG	C + FA60	MS + PG	MS + FA30	MS + FA60
**Body weight (g)**	460.3 ± 3.7	463.2 ± 5.6	465.1 ± 8.6	449.1 ± 8.1	452.1 ± 9.4
**Liver wet weight/B.W. (mg/g)**	28.6 ± 0.6	28.7 ± 0.4	39.8 ± 2.0 *	30.9 ± 0.2 *^#^	29.7 ± 0.7 ^#†^
**Heart wet weight/ B.W. (mg/g)**	2.76 ± 0.02	2.79 ± 0.08	3.08 ± 0.04 *	3.07 ± 0.04 *	3.07 ± 0.02 *
**LV wet weight/ B.W. (mg/g)**	1.87 ± 0.14	1.93 ± 0.04	2.23 ± 0.04 *	2.16 ± 0.04 *	2.13 ± 0.02 *

C + PG, normal control rats received propylene glycol as a vehicle; C + FA60, normal control rats received ferulic acid 60 mg/kg; MS + PG, MS rats received propylene glycol; MS + FA30, MS rats received ferulic acid 30 mg/kg; MS + FA60, MS rats received ferulic acid 60 mg/kg; B.W., body weight. Values are expressed as mean ± standard error of the mean (SEM) (*n* = 10/group). * *p* < 0.05 *vs.* C + PG; ^#^ *p* < 0.05 *vs.* MS group; ^†^ *p* < 0.05 *vs.* MS with FA 30 mg/kg.

### 3.2. Effect of FA on Fasting Blood Glucose and Oral Glucose Tolerance Test

HCHF diet was associated with a significant increase in FBG levels, AUC for the oral glucose tolerance test, fasting serum insulin and HOMA-IR scores when compared to control rats at the end of 16 weeks. The changes indicate an impaired glucose tolerance in the HCHF rats. FA treatment (30 and 60 mg/kg) significantly prevented these changes and alleviated the insulin resistant state in a dose-dependent manner. FA had no hypoglycemic effect and did not alter the glucose tolerance test results in control rats (Table 3).

**Table 3:** Effect of FA on metabolic variables and inflammatory cytokine.

Variables	C + PG	C + FA60	MS + PG	MS + FA30	MS + FA60
**FBG (mg/dL)**	86.7 ± 1.4	87.2 ± 1.7	117.0 ± 3.4 *	100.4 ± 0.9 *^#^	93.4 ± 1.2 *^#†^
**AUC (mg/dL/120 min)**	14,597 ± 137	14,455 ± 433	17,609 ± 263 *	16,808 ± 273 *^#^	15,840 ± 248.4 *^#†^
**HOMA-IR**	1.73 ± 0.05	1.68 ± 0.67	17.85 ± 3.2 *	4.45 ± 0.44 *^#^	2.91 ± 0.26 *^#†^
**Cholesterol (mg/dL)**	58.1 ± 3.2	57.7 ± 2.2	87.6 ± 2.0 *	69.9 ± 1.3 *^#^	63.5 ± 4.2 *^#^
**Triglycerides (mg/dL)**	38.40 ± 6.15	36.14 ± 5.83	78.29 ± 3.96 *	50.60 ± 2.91 *^#^	49 ± 2.89 *^#^
**HDL-C (mg/dL)**	34.32 ± 1.54	34.33 ± 0.95	19.07 ± 1.55 *	33.29 ± 0.97 *^#^	33.50 ± 1.49 *^#^
**Plasma TNF-α (pg/mL)**	27.77 ± 3.15	25.83 ± 3.36	173.14 ± 16.30 *	52.98 ± 8.90 *^#^	42.02 ± 3.84 *^#^

C + PG: normal control rats received propylene glycol as a vehicle; C + FA60: normal control rats receiving ferulic acid 60 mg/kg; MS + PG: MS rats receiving propylene glycol; MS + FA30: MS rats receiving ferulic acid 30 mg/kg; MS + FA60: MS rats receiving ferulic acid 60 mg/kg. Values are expressed as mean ± standard error of the mean (SEM) (*n* = 10/group). * *p* < 0.05 *vs.* C + PG; ^#^ *p* < 0.05 *vs.* MS group; ^†^ *p* < 0.05 *vs.* MS with FA 30 mg/kg. FBG, fasting blood glucose; AUC, area under the curve; HOMA-IR, homeostasis model assessment-estimated insulin resistance; HDL-C, high density lipoprotein-cholesterol; TNF-α, tumor necrosis factor-α.

### 3.3. Effect of FA on Lipid Profile and Plasma TNF-α

The HCHF diet induced significant increases in plasma total cholesterol, triglycerides and a significant decrease in HDL-cholesterol when compared with the control group. FA (30 and 60 mg/kg) significantly prevented the increase in plasma triglycerides, total cholesterol and decrease in plasma HDL-C. The values of these variables in normal controls-treated with FA did not differ from control values (C + PG, Table 3). A chronic inflammatory state is one of the important characteristics of metabolic syndrome. Plasma TNF-α was markedly elevated in MS rats and treatment with FA in HCHF animals largely suppressed release of the inflammatory cytokine (Table 3).

### 3.4. Effect of FA on Blood Pressure

**Figure 2:**
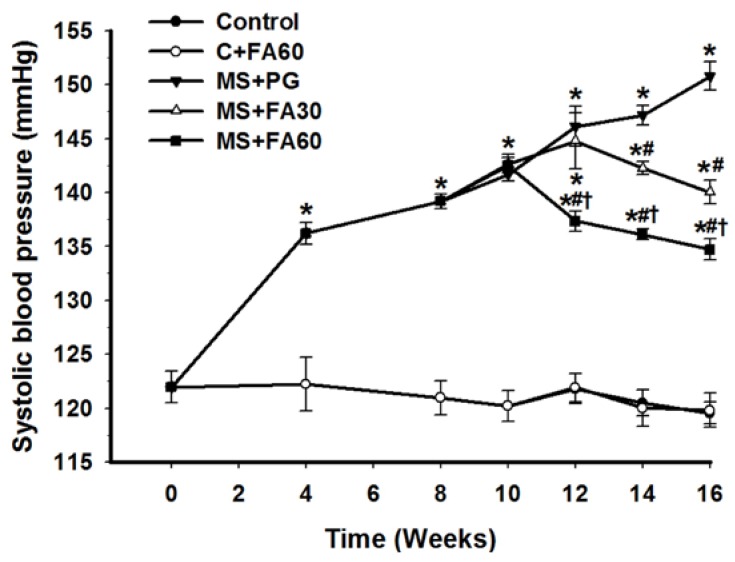
Effect of FA on systolic blood pressure in high carbohyrate and high fat (HCHF) induced high blood pressure. Normal control rats plus propylene glycol (solid circles), normal control rats treated with ferulic acid 60 mg/kg (open circles), MS rats plus propylene glycol (solid inverted triangles), MS rats treated ferulic acid 30 mg/kg (open triangles), MS rats treated with ferulic acid 60 mg/kg (solid squares). Each point represents the mean ± standard error of the mean (SEM) (*n* = 10/group). * *p* < 0.05 *vs.* C + PG; ^#^ *p* < 0.05 *vs.* MS group; **^†^** *p* < 0.05 *vs.* MS with FA 30 mg/kg. FA, ferulic acid; MS, metablic syndrome; C + PG, normal control rats receiving ferulic acid.

Metabolic syndrome is characterized by an elevation of blood pressure. The HCHF diet induced an increase in SBP (Figure 2) which was significant when compared to controls within 4 weeks of feeding and progressively increased throughout the 16 weeks of the study period. FA, administered from week 10 through week 16, significantly prevented the increased SBP when compared to the MS (MS + PG) group. The antihypertensive effect was evident within 2 and 4 weeks following the adminsitration of high and low doses of FA, respectively, and the antihypertensive effect was demonstrated in a dose-dependent manner. In contrast, FA when administered to control rats fed a normal diet did not show any hypotensive or blood pressure lowering effect.

### 3.5. Effect of FA on Hemodynamic Parameters and Vascular Reactivity

HCHF diet fed rats showed the abnormalities of cardiovascular dynamics as demonstrated by the increase in SBP, mean arterial pressure (MAP), diastolic blood pressure (DBP) and HR when compared with controls (Table 4). These changes were associated with a decrease in HBF and increased HVR (Table 4). Increased heart rate in HCHF-fed rats could be the cause of blood pressure elevation. However, we found that heart rate contributed less to the development of hypertension than the peripheral vascular resistance, since the heart rate of the HCHF group increased by only 23%, whereas the hindlimb vascular resistance more than doubled with respect to the normal control values (Table 4). Interestingly, treatment with FA (30 and 60 mg/kg) significantly alleviated the changes in a dose-dependent manner when compared with MS rats.

**Table 4:** Effect of FA on hemodynamic parameters in MS rats.

Variables	C + PG	C + FA60	MS + PG	MS + FA30	MS + FA60
**SBP (mmHg)**	120.4 ± 1.4	120.7 ± 1.1	151.6 ± 2.0 *	140.6 ± 3.6 *^#^	132.7 ± 1.6 *^#†^
**DBP (mmHg)**	78.1 ± 1.7	75.6 ± 1.7	108.4 ± 1.9 *	97.7 ± 1.3 *^#^	89.4 ± 1.5 *^#†^
**MAP (mmHg)**	95.4 ± 1.6	94.4 ± 1.2	124.5 ± 1.9 *	117.3 ± 2.4 *^#^	109.0 ± 1.3 *^#†^
**HR (beat/min)**	344.7 ± 3.4	346.6 ± 8.8	424.0 ± 3.2 *	406.3 ± 10.3 *	383.3 ± 7.6 *^#†^
**HBF (mL/min/100 g tissue)**	6.3 ± 0.3	6.3 ± 0.2	4.0 ± 0.2 *	5.2 ± 0.1 *^#^	5.9 ± 0.2 ^#†^
**HVR (mmHg/mL/min/100 g tissue)**	15.14 ± 0.30	15.68 ± 0.30	31.73 ± 0.30 *	23.93 ± 0.30 *^#^	17.72 ± 0.30 *^#†^

C + PG: normal control rats received propylene glycol as a vehicle; C + FA60: normal control rats received ferulic acid 60 mg/kg; MS + PG: MS rats received propylene glycol; MS + FA30: MS rats received ferulic acid 30 mg/kg; MS + FA60: MS rats received ferulic acid 60 mg/kg. Values are expressed as mean ± standard error of the mean (SEM) (*n* = 10/group). * *p* < 0.05 *vs.* C + PG; ^#^ *p* < 0.05 *vs.* MS group; **^†^** *p* < 0.05 *vs.* MS with FA 30 mg/kg; FA, ferulic acid; MS, metabolic syndrome; SBP, systolic blood pressure; DBP, diastolic blood pressure; MAP, mean arterial blood pressure; HR, heart rate; HBF, hindlimb blood flow; HVR, hindlimb vascular resistance.

**Figure 3:**
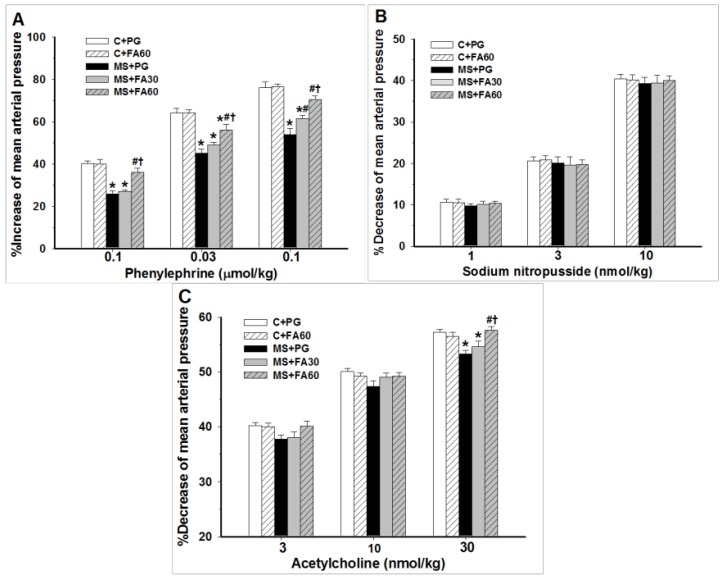
Effect of FA on vascular responses in MS rats. The blood pressure response in rats to (**A**) phenylephrine-induced high blood pressure; (**B**) sodium nitroprusside-induced decreased blood pressure; and (**C**) acetylcholine-induced decreased blood pressure was assessed. The mean arterial pressures are presented as mean ± standard error of the mean (SEM) (*n* = 10/group); * *p* < 0.05 *vs.* C + PG; ^#^ *p* <0.05 *vs.* MS group; **^†^** *p* < 0.05 *vs.* MS with FA 30 mg/kg. C + PG: normal control rats received propylene glycol as a vehicle; C + FA60: normal control rats receiving ferulic acid 60 mg/kg; MS+PG: metabolic syndrome rats receiving propylene glycol; MS+FA30: metabolic syndrome rats receiving ferulic acid 30 mg/kg; MS+FA60: metabolic syndrome rats receiving ferulic acid 60 mg/kg. FA, ferulic acid; MS, metabolic syndrome.

Furthermore, MS rats showed diminished vascular responses to the vasoactive agents, Phe (Figure 3A) and ACh (Figure 3C) when compared with the normal control group. MS rats-treated with FA in a dose-dependent manner restored the vascular responsiveness by preventing the attenuation of the vasoconstrictive and vasodilatory effects of Phe and ACh (Figure 3A,C). In MS rats, the vascular response to SNP, an endothelium-independent vasodilator, was unchanged (SNP; Figure 3B) and was not altered following the treatment with FA.

### 3.6. Effect of FA on Oxidative Stress

**Figure 4:**
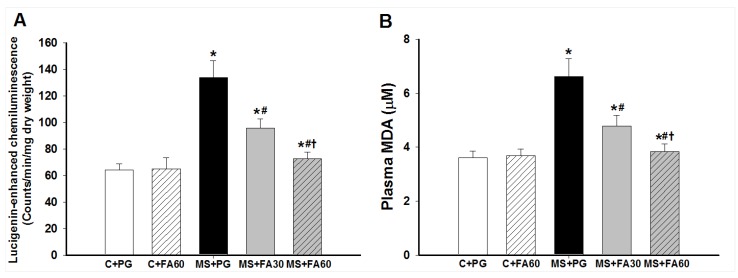
Effect of FA on superoxide and lipid peroxidation in MS rats. Superoxide production was measured from carotid strips dissected after the end of this study (**A**) and plasma samples were taken for assay of malondialdehyde (MDA) (**B**). Values are presented as mean ± standard error of the mean (SEM) (*n* = 10/group); * *p* < 0.05 *vs.* C + PG; ^#^ *p* < 0.05 *vs.* MS group; **^†^** *p* < 0.05 *vs.* MS with FA 30 mg/kg. C + PG: normal control rats received propylene glycol as a vehicle; C + FA60: normal control rats received ferulic acid 60 mg/kg; MS + PG: MS rats received propylene glycol; MS + FA30: MS rats received ferulic acid 30 mg/kg; MS + FA60: MS rats received ferulic acid 60 mg/kg. FA, ferulic acid; MDA, malondialdehyde.

Excess production of ROS is one of the main factors contributing to lipid peroxidation and oxidative damage in metabolic syndrome. In this study, we assessed vascular oxidative status by measurement of superoxide production in carotid arteries and oxidative products of lipid peroxidation in plasma. Figure 4A shows that vascular superoxide production from carotid strips was significantly higher in MS rats than in the control groups. Plasma MDA levels were significantly greater in MS rats than controls (Figure 4B). Administration of FA (30 and 60 mg/kg) significantly prevented the HCHF-induced increase in vascular superoxide production and plasma MDA in a dose dependent manner (Figure 4A,B). FA treatment did not alter basal superoxide formation or any of the oxidative stress markers in normal control rats.

### 3.7. Effect of FA on Nitric Oxide Formation

**Figure 5:**
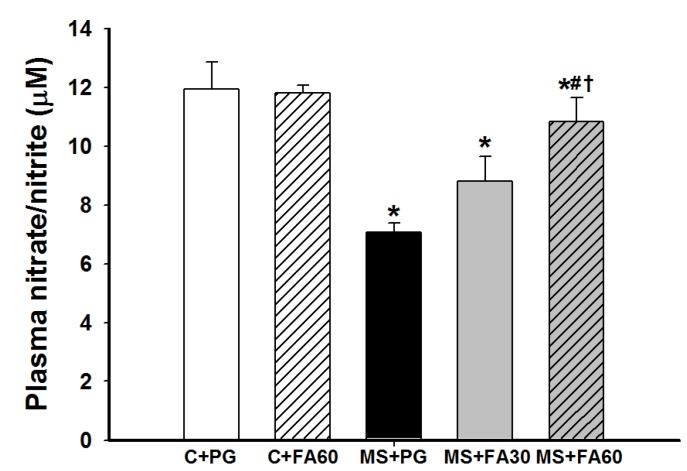
Effect of FA on plasma nitrate/nitrite levels in MS rats. Values are presented as mean ± standard error of the mean (SEM) (*n* = 8/group); * *p* < 0.05 *vs.* C + PG; ^#^ *p* < 0.05 *vs.* MS group; **^†^** *p* < 0.05 *vs.* MS with FA 30 mg/kg. FA, ferulic acid; MS, metabolic syndrome.

NO released from vascular tissues plays critical roles in the vascular response, changes in pressure and flow, and cardiovascular protection [29]. The plasma nitrate/nitrite ratio, representing NO metabolites, in MS rats was significantly lower than in the control groups. FA prevented the attenuation of plasma nitrate/nitrite levels when compared with the HCHF group not treated with FA. FA treatment did not affect plasma NO in control rats (Figure 5).

### 3.8. Effect of FA on Arterial Histomorphometry

**Figure 6:**
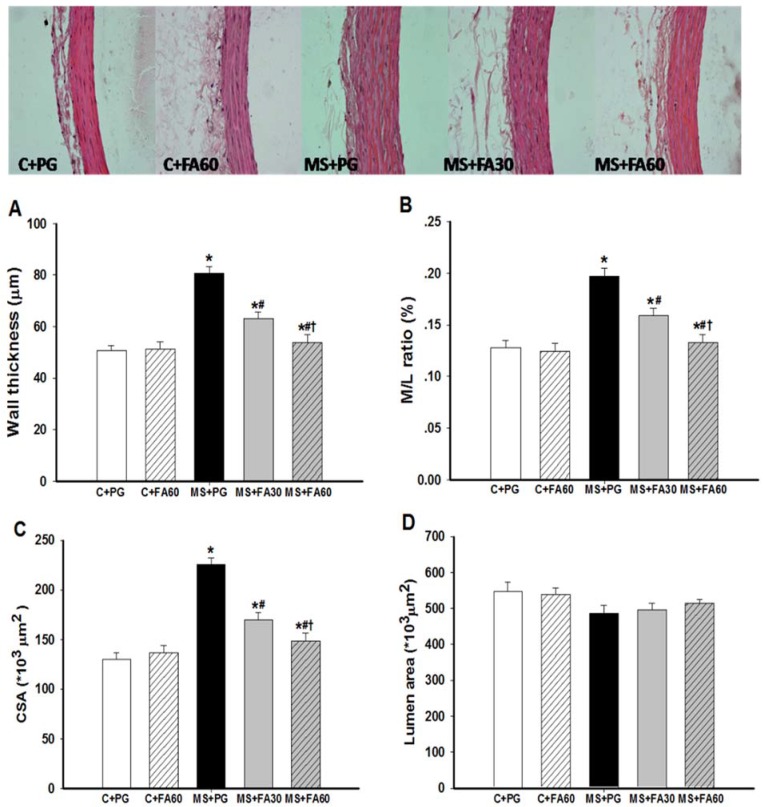
Effect of FA on vascular remodeling of mesenteric arteries in MS rats. Representative photomicrographs of the mesenteric arteries (×400) stained with hematoxylin and eosin are shown and morphometric analysis was performed for (**A**) the wall thickness; (**B**) media to lumen ratio (M/L); (**C**) cross-sectional area (CSA) of the media layer; (**D**) lumen area. Values are presented as mean ± standard error of the mean (SEM) (*n* = 6/group); * *p* < 0.05 *vs.* C + PG; ^#^ *p* < 0.05 *vs.* MS group; **^†^** *p* < 0.05 *vs.* MS with FA 30 mg/kg. FA, ferulic acid; MS, metabolic syndrome.

Figure 6 illustrates the histological changes in mesenteric arteries from the various experimental groups. Vascular wall thickness, M/L ratio and CSA were significantly increased in the HCHF group. However the luminal cross sectional area remained unchanged (Figure 6D). The histological changes in the vessel medial layer were indicative of hypertrophic vascular remodeling. These results show that, chronic consumption of HCHF induced vascular remodeling and that treatment with FA (30 and 60 mg/kg) attenuated this remodeling in a dose dependent manner as indicated by significantly reduced vascular wall thickness, M/L ratio and CSA (Figure 6A–C).

### 3.9. Effect of FA on Arterial Protein Expression of eNOS and p47^phox^

**Figure 7:**
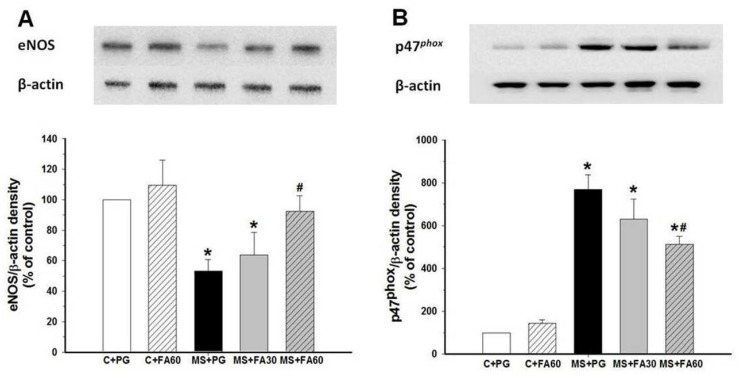
Effect of FA on eNOS protein **(A)** and p47^phox^ protein **(B)** expression in aortas of MS rats. Data are presented as a percentage of normal controls values and expressed as mean ± standard error of the mean (SEM). Data obtained from at least five different experiments. * *p* < 0.05 *vs.* C + PG, ^#^ *p* < 0.05 *vs.* MS group. FA, ferulic acid; eNOS, endothelial nitric oxide synthase; MS, metabolic syndrome; C + PG, normal control rats received propylene glycol.

There were changes in NO and O_2_^•−^ production in MS rats which were largely prevented by FA. However, it is not clear whether these changes were associated with up-regulation and/or down-regulation of eNOS and NADPH oxidase enzymes. Therefore, Western blot analysis was performed to examine the expression levels of eNOS and NADPH oxidase subunit p47^phox^ in the aorta. When comparing MS rats with controls, we observed a significant decrease in eNOS expression (Figure 7A), whereas the protein expression of p47^phox^ was significantly increased (Figure 7B). Thus, FA administration (60 mg/kg) prevented the reduction of eNOS and the increased p47^phox^ expression. However, FA treatment in normal control rats did not cause any significant changes in the protein expression of eNOS and p47^phox^ subunit (Figure 7A,B).

## 4. Discussion

In the present study we have demonstrated that rats fed with the HCHF diet for 16 weeks developed the signs of metabolic syndrome including hyperglycemia, insulin resistance, dyslipidemia, high blood pressure, vascular remodeling, oxidative stress and inflammation. The beneficial effect of oral supplementation of FA in MS rats for six weeks was evaluated and it was found that FA could reverse almost all the deleterious changes in these animals. The protective effects resulted from the restoration of insulin sensitivity, and normalization of blood pressure and vascular responsiveness, whereas the underlying mechanism may be the suppression of oxidative stress by downregulation of NADPH oxidases, inhibition of inflammatory cytokine and maintenance of nitric oxide availability.

Animals fed with HCHF diet did not gain weight in comparison with control rats. However, organ weight, particularly that of the liver was increased. This is consistent with other reports using a similar HCHF rat model which found liver inflammation and steatosis, together with cardiac hypertrophy [30]. In this study, FA supplementation was started at week 10, which was after hypertension had already developed. FA was associated with decreased blood pressure, but not decreased heart and left ventricular weights. This suggests that cardiac hypertrophy having resulted from remodeling would take more time to regress to normal. Moreover, FA also decreased the liver weight. The exact mechanism of this effect is not known and needs further study. However, since liver cells can change rather rapidly due to their high rate of division when they are stimulated, the reduced liver weight in FA-supplemented rats is probably due to the suppression of liver cells’ proliferation. FA reduces oxidative stress and insulin resistance, thereby, normalizing lipid metabolism and suppressing inflammation, leading to an alleviation of the fatty liver and also preventing the changes in arterial morphology and function.

Insulin resistance is a hallmark of metabolic syndrome and type 2 diabetes. The elevation of FBG and plasma insulin resulting in an increase of HOMA-IR is largely alleviated by FA. This suggests that FA may have an insulin sensitizing effect, which it is consistent with previous reports of the anti-diabetic action of FA in type 2 diabetic mice [11]. The restoration of insulin sensitivity, in turn, may account for the improvement in lipid profile, *i.e.*, reduction in hypercholesterolemia and hypertriglyceridemia and increased HDL-C. It should be noted that the HCHF diet-induced MS is in part associated with the release of inflammatory cytokines, *i.e.*, TNF-α in the present study. The release of inflammatory cytokines is known to be a very strong inducer of insulin resistance [31]. In a recent study we reported the inflammatory cytokine-induced insulin resistance in human hepatocellular liver carcinoma cell line (HepG2), whereas suppression of its signaling pathway restored insulin sensitivity [32]. The suppression of plasma TNF-α by FA found in the present study may significantly contribute to the insulin sensitizing effect.

The elevation of blood pressure is another feature of the metabolic syndrome state. Patients with MS and type 2 diabetes are frequently afflicted with cardiovascular complications, for instance: hypertension, coronary heart disease and ischemic stroke [33]. The good control of blood pressure in hypertensive and diabetic patients is known to benefit cardiovascular outcomes [34]. The etiology of hypertension remains poorly understood. However, oxidative stress in association with a chronic inflammatory state plays a major role in the modulation of vasomotor tone and vascular remodeling [29]. We observed an impaired vascular response and high blood pressure in MS rats, which was associated with vascular dysfunction, suppression of cytoprotection plasma nitric oxide and vascular eNOS expression, and an increase of p47phox expression. Ferulic acid has been shown to be a potent antioxidant *in vitro* and *in vivo* by up-regulating the strongly cytoprotective enzyme, heme oxygenase-1(HO-1), heat shock protein 70 (HSP70) and Protein kinase B (Akt), as well as suppressing oxidant and inflammation generation by down-regulation of cyclooxygenase-2 (COX-2) [4,7]. Moreover, FA was shown to inhibit angiotensin converting enzyme (ACE) activity [8]. In the HCHF diet-induced high blood pressure and vascular dysfunction reported here, the antioxidant, anti-inflammation and ACE inhibition of FA could account for the antihypertensive and vascular protective effects. It should be noted that FA did not lower blood pressure in normal rats, suggesting that its blood pressure lowering effect is observed only under pathological conditions.

Long standing insulin resistance in metabolic syndrome and diabetes, as well as hypertension cause macrovascular changes like atherosclerosis and eventually leads to cardiovascular complications and ischemic stroke [33]. In this study, there was a thickening of the media of the mesenteric arterial wall, which may be due to the proliferation and migration of smooth muscle cells and or accumulation of extracellular matrix [35]. The thickened media may lead to increased arterial stiffness, a feature associated with the development of atherosclerosis. Treatment with FA attenuated the vascular changes in HCHF diet rats so that the vessels appeared normal. Previous reports have shown that FA can reduce inflammatory cell infiltration and collagen deposition in kidney and heart tissue [36]. Moreover, the effects of angiotensin II and oxidants which play a critical role in vascular damage could be abolished by FA [37]. Altogether, the prevention of vascular remodeling could be very important aspect of the way in which FA may prevent cardiovascular complications in the chronic metabolic syndrome state.

The antioxidant effects of FA may be due not only to free radical scavenging activity which was observed as inhibition of superoxide and MDA formation, but may also include suppression of p47^phox^ expression, the enzymes generating reactive oxygen species. The restoration of eNOS expression may be a consequence of suppression of oxidant formation, as FA had no effect on NO and eNOS levels in control animals. 

## 5. Conclusions

The present study demonstrates that the HCHF diet induces metabolic syndrome-like signs in rats and that this was associated with oxidative stress, inflammation and vascular remodeling. Oral supplementation of FA ameliorates HCHF-induced MS, improves insulin sensitivity, lipid profiles and vascular endothelial function, decreases blood pressure, and reduces oxidative stress and inflammation. These therapeutic effects of FA may be due to its antioxidant and anti-inflammatory properties. This study provides evidence of the health benefit of FA consumption.
